# (*CF*)^2^ architecture: contextual collaborative filtering

**DOI:** 10.1007/s10791-018-9332-3

**Published:** 2018-05-16

**Authors:** Dennis Bachmann, Katarina Grolinger, Hany ElYamany, Wilson Higashino, Miriam Capretz, Majid Fekri, Bala Gopalakrishnan

**Affiliations:** 1 Department of Electrical and Computer Engineering, Western University, London, ON Canada; 2 Department of Computer Science, Suez Canal University, Ismailia, Egypt; 3 Pelmorex Media, Oakville, ON Canada

**Keywords:** Recommender system, Collaborative filtering, Context awareness, Local learning

## Abstract

Recommender systems have dramatically changed the way we consume content. Internet applications rely on these systems to help users navigate among the ever-increasing number of choices available. However, most current systems ignore the fact that user preferences can change according to context, resulting in recommendations that do not fit user interests. This research addresses these issues by proposing the $({ CF})^2$ architecture, which uses local learning techniques to embed contextual awareness into collaborative filtering models. The proposed architecture is demonstrated on two large-scale case studies involving over 130 million and over 7 million unique samples, respectively. Results show that contextual models trained with a small fraction of the data provided similar accuracy to collaborative filtering models trained with the complete dataset. Moreover, the impact of taking into account context in real-world datasets has been demonstrated by higher accuracy of context-based models in comparison to random selection models.

## Introduction

In everyday life, it is not uncommon to rely on recommendations by friends or family to decide on a restaurant for dinner. During the years, many publications around the globe have specialized in providing people with lists of recommendations on all kinds of topics. Nowadays, Internet applications have turned to recommender systems to help users navigate among the ever-increasing number of available choices.

Recommender systems can be broadly categorized as collaborative, content-based, or hybrid (Kotsogiannis et al. 2017). Collaborative recommendation resembles word-of-mouth communication, in which the opinions of others are used to determine the relevance of a recommendation. In this case, a collaborative recommender system uses the ratings provided by its users either to recommend an interesting item or to identify like-minded users. Likewise, content-based recommendation focusses on using the content of an item to assert its relevancy. The hybrid category is reserved for those systems that use both techniques when deliberating on a recommendation.

Extensive work has been done to develop new collaborative recommender methods (Su 2015; Shi et al. 2014; Pirasteh et al. 2014). Nevertheless, in many applications, these methods do not suffice because context is not taken into account. In fact, if only items and users are used to provide recommendations, it would be safe to assume that a travel agency is making a proper recommendation if it suggests a ski package to someone who is interested in skiing. However, this probably would not be a wise idea if the recommendation were given in the peak of summer. Therefore, it is important to incorporate contextual information into the recommendation process.

With the proliferation of smart phones, smart watches, and other smart devices, applications have access to more and more contextual information from their users, and yet recommender systems fail to use this contextual information when giving recommendations. One way of addressing this issue is to create new models that can incorporate user context and thereby improve the quality of their recommendations (Adomavicius and Tuzhilin 2011; Panniello et al. 2009; Colombo-Mendoza et al. 2017).

The difficulty of incorporating user context into new models presents a challenge because it adds new dimensions to the model. Moreover, the volume and speed at which contextual data are generated become a challenge for training new models. More specifically, the processing power required to train a model using such large datasets is enormous (Liu and Motoda 2001).

This paper leverages local learning techniques to propose the *Context Filtering for Collaborative Filtering* $({ CF})^2$ architecture. $({ CF})^2$ relies on existing collaborative filtering techniques but adds contextual awareness to recommendations. Given that existing collaborative models are unaware of user context when making recommendations, the architecture uses available contextual information as filtering criteria for a local learning technique, which attempts to adjust the capacity of the training system locally to the properties of the training set in each area of the input space (Bottou and Vapnik 1992). By rearranging the data in this way, each generated model represents a context, and these models are used to generate contextual recommendations. The context could potentially just be added as a new dimension into the recommendation problem, but this would increase computation time. Using context as a splitting criteria, $({ CF})^2$ builds several smaller models instead of one large one what is especially beneficial with large data sets.

The proposed approach was evaluated in the “Find good items” task (Gunawardana and Shani 2009). The context-based filtering has been implemented using the proposed architecture and evaluated on two large real-world datasets: the first one with over 130 million and the second one with over 7 million unique samples. The studies demonstrate the proposed $({ CF})^2$ architecture and show that the context-based filtering outperforms traditional global models in terms of accuracy. In addition, local models created using random sampling were compared to contextual dataset reduction to examine the impact of context in large datasets. The results demonstrated that use of contextual information greatly outperformed random selection in accuracy.

The remainder of this paper is organized as follows. Section 2 provides background concepts. Section 3 presents related work. Section 4 gives an overview of $({ CF})^2$ and its components. Section 5 presents an evaluation of $({ CF})^2$. Section 6 presents the conclusions of this work and suggestions for future work.

## Background

This section introduces and discusses the concepts of collaborative filtering, local learning, and contextual inference, which are the foundation for understanding $({ CF})^2$.

### Collaborative filtering

Collaborative filtering (*CF*) was the first technique used by a recommender system and is also considered to be the most popular and widely implemented (Ricci et al. 2015). Examples of popular Web sites that use this technique are *Amazon*^1^ (Linden et al. 2003), *TiVo*,^2^ and *Netflix*.^3^

*CF* provides recommendations based on the opinions of others who share the same interests as the user (Lu et al. 2015). These opinions are often represented as the ratings matrix *R* (Aggarwal 2016b). This matrix is an $m\times n$ matrix containing *m* users and *n* items. Hence, the rating of user *u* for item *i* is given by $r_{{ ui}}$. Because in any recommender system the number of ratings obtained is usually very small compared to the number of ${ users} \times { items}$, the matrix *R* is often sparse (Bindu et al. 2017).

The approach adopted by *CF* methods is that these missing ratings can be guessed because the observed ratings are often highly correlated across various users and items (Aggarwal 2016a).

This method has cold-start and sparsity problems and is also limited by the fact that ratings can be predicted only if users have rated common items (Son 2016). Data reduction techniques appear to be a promising research direction to solve this problem (Adomavicius and Tuzhilin 2005). *CF* also has the advantage of using an approach that enables it to obtain meaningful relations between users or items that are not directly connected (Ricci et al. 2015).

### Local learning

Local learning algorithms attempt to divide the training set into several local clusters to capture more effectively the properties of each input space neighbourhood (Piegat and Pietrzykowski 2016). This results in creating separate local models for each cluster (Al-Jarrah et al. 2015; L’Heureux et al. 2017).

Local learning is based on the assumption that large training datasets are very rarely evenly distributed in the input space (Bottou and Vapnik 1992). Moreover, current machine learning systems are not inherently efficient or scalable enough to deal with large data volumes, and therefore a growing fraction of data remain unexplored or underexploited (Al-Jarrah et al. 2015). Hence, local learning is considered a suitable approach for machine learning algorithms that use large data volumes (Al-Jarrah et al. 2015).

As defined by Bottou and Vapnik (1992), local learning can be accomplished by performing a simple local algorithm for each testing pattern:

1. Select the training samples located in the vicinity of the test pattern. The vicinity refers to the test pattern neighbourhood and includes other samples close to the test pattern.
2. Train the model using only these samples.
3. Apply the resulting model to the test pattern.

Recent studies have shown that local learning yields results far superior to a global learning strategy, especially on datasets that are not evenly distributed (Ansell et al. 2017; Lughofer and Pratama 2017; Pratama et al. 2016).

In addition, for computationally intensive algorithms, it is faster to find solutions for *k* problems of size *m* / *k* than for one problem of size *m* (Grolinger et al. 2014).

### Contextual inference

**Fig. 1:**
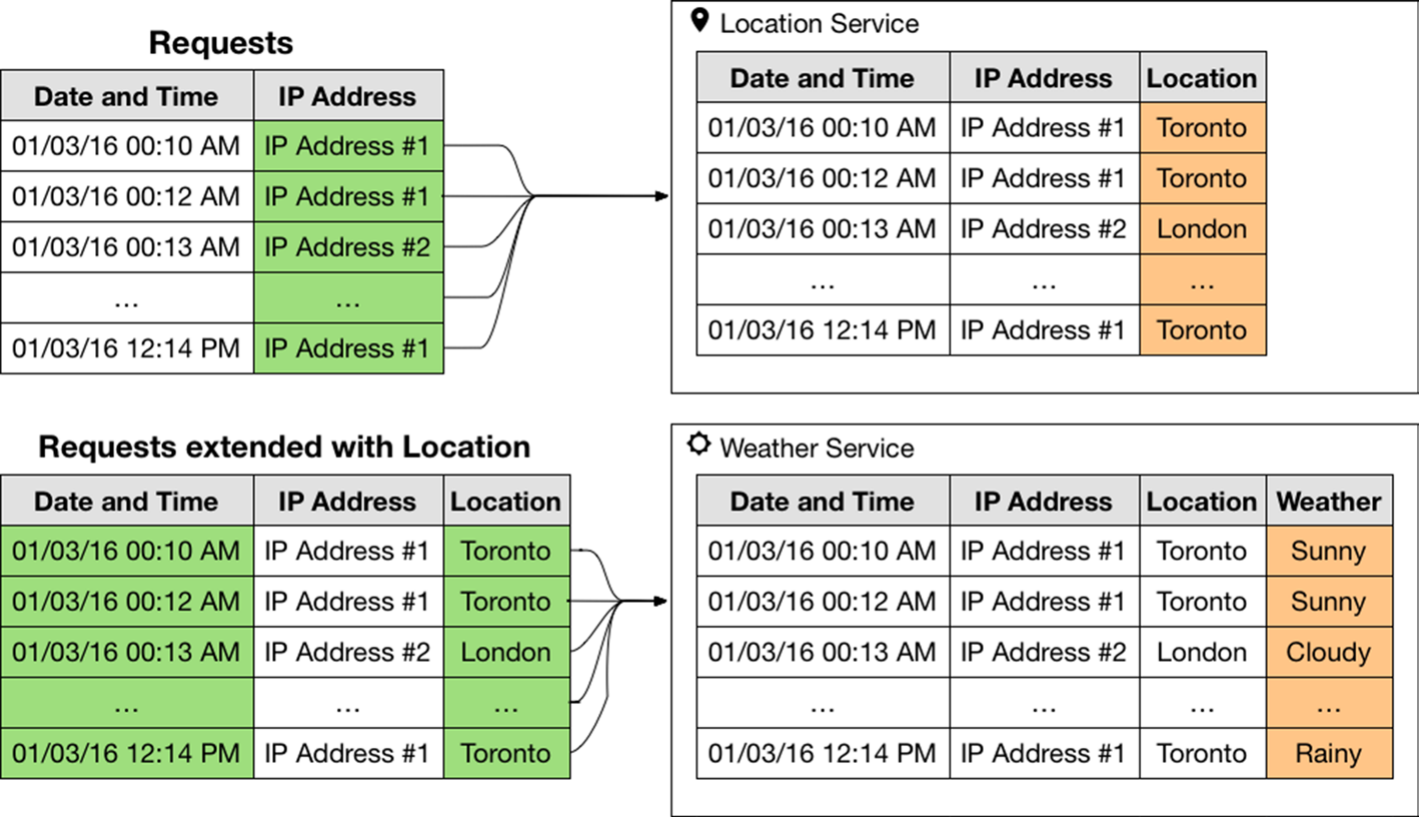
Example of contextual inference for location and weather conditions

Contextual inference is a process that uses inference rules or external knowledge to enrich existing datasets. An example of this process is illustrated in Fig. 1, which shows the process of extending a request containing an IP address and a time to contain the location and the weather condition at the time of the request. This is achieved by first querying a geolocation service to obtain the approximate geographic location of the client and then using this location and the time of the request to query a weather service to obtain the weather conditions.

Although this example uses external data or services to extend the contextual attributes, this may not be necessary. On some occasions, these transformations can be done using internal logic, like the classification of a given day as a weekday or a weekend. Therefore, this process enables contextual attributes that may look uninteresting at first, but can be extended to provide powerful contextual meaning to data.

## Related work

Related work is divided into three sub-sections. The first covers local learning and provides a review of how this technique has been used, the second section describes the use of contextual information in recommender systems, and the third section reviews architectures for contextual recommender systems.

### Local learning

Several studies have used local learning to achieve simplified local models and increased precision. Piegat and Pietrzykowski (2016) presented a new version of the mini-model method. In their work, the authors extended the mini-model method to include local dimensionality reduction, leading to simpler local models and increased precision.

The work conducted by Domeniconi et al. (2007) also aimed to perform dimensionality reduction. They tackled the curse of dimensionality by using local feature weighting to discover clusters in input sub-spaces. Their method associated with each cluster a weight vector, whose values were then used to capture the relevance of features within the corresponding cluster.

Wu and Schölkopf (2006) presented a local learning approach for clustering. Their idea was that an adequate clustering result should have the property that the cluster label of each data point can be well predicted based on its neighbouring data and their cluster labels. Relaxation and eigen-decomposition techniques were used to solve this problem. In addition, the authors provided a parameter selection method for the proposed clustering algorithm.

Chitta et al. (2015) proposed a sparse kernel *k*-means clustering algorithm that incrementally sampled the most informative points from the dataset using importance sampling and constructed a sparse kernel matrix using these sampled points. This sparse kernel matrix was then used to perform clustering and obtain cluster labels. This combination of sampling and sparsity reduces both the running time and the memory complexity of kernel clustering.

Zhou et al. (2016) proposed a global and local structure-preserving sparse sub-space learning model for unsupervised feature selection. Their model can perform feature selection and sub-space learning simultaneously.

Reviewed studies (Piegat and Pietrzykowski 2016; Domeniconi et al. 2007; Wu and Schölkopf 2006; Chitta et al. 2015; Zhou et al. 2016) focussed on ways of adapting existing models to simplify them and improve their accuracy while also performing dimensionality reduction. Nevertheless the models presented were static and could not be directly extended to other recommender models. In contrast, our work focusses on contextual information that is explicitly available in the dataset or can be inferred to perform clustering. Hence, by using context, the properties of local learning can be applied to existing recommender algorithms.

### Contextual information in recommender systems

Several studies have focussed on enhancing recommendation accuracy by using contextual information gathered from user interactions. Costa and Manzato (2016) proposed to improve prediction accuracy by incorporating various types of feedback into the recommendation process. In their work, each type of feedback is trained using a distinct recommender algorithm, and the results are unified into a final score.

Wei et al. (2017) tackled the cold-start problem that is common in recommender systems by integrating the *CF* approach with machine learning algorithms. Their approach uses a deep learning neural-network model to improve overall recommendation accuracy for cold-start situations. The authors focussed only on time contexts.

Capdevila et al. (2016) used geo-location information to improve the accuracy of recommendations in the context of a location-based social network. Using mining techniques, the proposed recommender system uses geolocated time-referenced reviews of venues to recommend locations to a user based on current geographic location.

In contrast to these studies (Costa and Manzato 2016; Wei et al. 2017; Capdevila et al. 2016), the present research proposes an extensible architecture that can leverage contextual information to create contextualized local models. This enables recommendations using any kind of contextual information. By using local learning with contextual information to generate the models, only the ratings made in the same context as the target prediction are used. Moreover, by using a pre-filtering approach on the training set, any *CF* model can be used to generate the recommendations.

Adomavicius and Tuzhilin (2011) argued the importance of contextual information in recommender systems, discussed the notion of context and how it can be modeled. The described pre-filtering paradigm uses contextual information to select data for recommendations. Similarly, Adomavicius et al. (2005) presented a multidimensional recommendation model which also uses context information such as time to carry out filtering. Likewise, our study uses context to select data for building the contextual models; however, we also propose an architecture for building such systems. Moreover, Adomavicius and Tuzhilin (2011) focused on reviewing approaches for modeling the context without experimental evaluation. Adomavicius et al. (2005) evaluated the proposed approach on the dataset of around 1500 records specifically created for this purpose through Web survey on movies. In contrast, our evaluation was performed on a real-world dataset containing information about website visitors clicks (over 130 million records in case study 1 and over three million records in case study 2). Evaluation with real-world datasets is important as it better represents the scenario in which recommendation system will be used.

Palmisano et al. (2008) also incorporate pre-filtering in their approach: the customers are first clustered, and then pre-filtering is applied to incorporate context. The evaluation is performed on two datasets: an artificial set and a real-world dataset set artificially augmented to increase size. In contrast, our study does not use clustering, proposes an architecture, and evaluates on real-world datasets.

### Architectures for contextual recommender systems

Eirinaki et al. (2018) surveyed large-scale recommender systems built for social networks; they outlined several challenges that might have a negative impact on such systems including data volume, variety, and volatility. To handle the issue of data volume, they suggested using various technologies such as Hadoop, Spark and MapReduce to process the data in parallel and to perform machine learning tasks such as predictions. Our work proposes a local learning-based architecture that can efficiently analyze large datasets by splitting them into smaller sets using different contextual information. The evaluation of the proposed architecture outperforms the traditional solutions.

Yao et al. (2015) proposed a graph-based recommendation framework that constructs a multi-layer context graph from implicit feedback data and then executes ranking algorithms on this graph to produce context-aware recommendations. The proposed graph models the interactions between users and items and incorporates a variety of contextual information into the recommendation process.

De Pessemier et al. (2014) described a framework to detect the current context and user activity by analyzing data retrieved from various sensors available on mobile devices. On top of this framework, a recommender system was built to offer users personalized content consisting of relevant information such as points of interest, train schedules, and tourist information.

Unlike the works of Yao et al. (2015) and De Pessemier et al. (2014), our architecture can leverage contextual information to create smaller local models using different contextual information. Moreover, our case studies demonstrate the proposed architecture on large real-world datasets.

## $(\hbox {CF})^2$ architecture

**Fig. 2:**
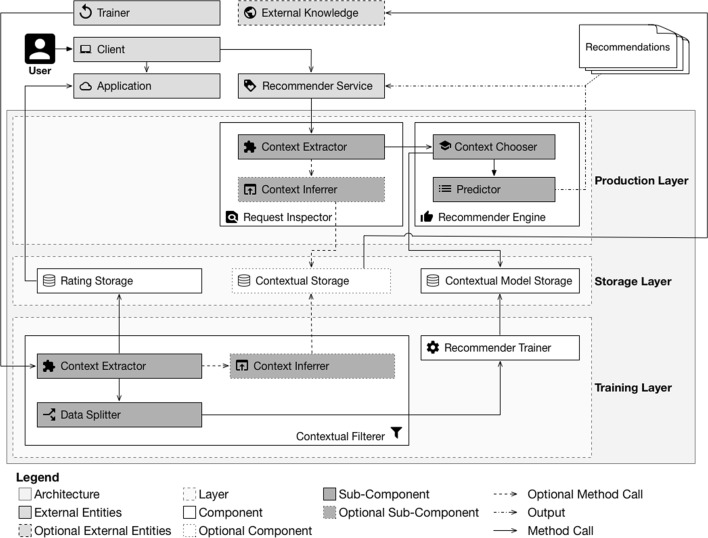
Context filtering for collaborative filtering $({ CF})^2$ architecture

**Fig. 3:**
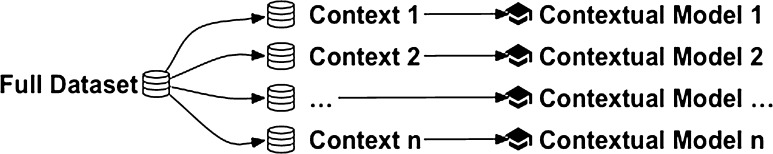
Dataset divided into smaller subsets based on contextual attributes

This section introduces the context filtering for collaborative filtering $({ CF})^2$ architecture, illustrated in Fig. 2. It uses embedded contextual attributes available in the datasets as filtering criteria to apply local learning techniques. Each resulting subset is then used to train contextual *collaborative filtering* models that are trained using only the portion of the rating data that matches the contextual criteria. This is exemplified in Fig. 3, where a full dataset is divided into smaller subsets based on contextual criteria.

$({ CF})^2$ operates in two phases: a *training phase* and a *production phase*. Each of these phases is represented as a layer in the architecture. Moreover, an additional third layer, called the *storage layer*, is used to handle rating data and contextual information retrieval. This layer is also responsible for storing the trained models. The three layers ensure better separation of concerns and provide stronger decoupling between the phases.

The following subsections describe the architectural details of the proposed $({ CF})^2$.

### External entities

The external entities are the components that $({ CF})^2$ must interact with to provide meaningful recommendations to its users, but are not part of the $({ CF})^2$ architecture itself. As illustrated in the upper part of Fig. 2, they include: trainer, client, application, external knowledge and recommender service.

The *client* is a software application responsible for capturing ratings given by a user and submitting them to an *application*. The *application* is the service that stores the ratings given by a *client*. The *client* may also be involved in obtaining recommendations from the *recommender service*.

The *recommender service* is the service responsible for processing requests issued by *clients* when they are in need of recommendations. Requests issued to this service must be accompanied by a client identifier, an identifier for the item for which the requesting party wants to obtain recommendations, and a list of contextual attributes. The request must include the item identifier because the user is looking for recommendations similar to a specific item.

The *trainer* is the service responsible for initiating the training phase. This service can be manually invoked by a system administrator or periodically invoked by a time-based job scheduler. The *external knowledge* is the service that holds the additional contextual information used by the *contextual storage* component.

The only artifact generated by $({ CF})^2$ is the list of recommendations that are returned when requested by the *client*.

### Storage layer

This layer contains all the components required by $({ CF})^2$ to access data in a standard manner. Because the data used by $({ CF})^2$ may be stored by third-party applications, this layer provides a set of components that serves as abstraction to these datasets.

The *rating storage* ensures a standardized interface to access the rating dataset stored in the *application*. This interface will be used during the training phase to provide access to these ratings.

The *contextual storage* component ensures a standardized interface to access the datasets that can provide additional contextual information to the data. $({ CF})^2$ can use more than one service to provide external contextual data. Hence, this component implements a method for each of these services to abstract the peculiarities of each external service.

The use of external contextual attributes is achieved by matching embedded contextual attributes available in the *rating storage* component with those provided by the *contextual storage* component.

**Fig. 4:**
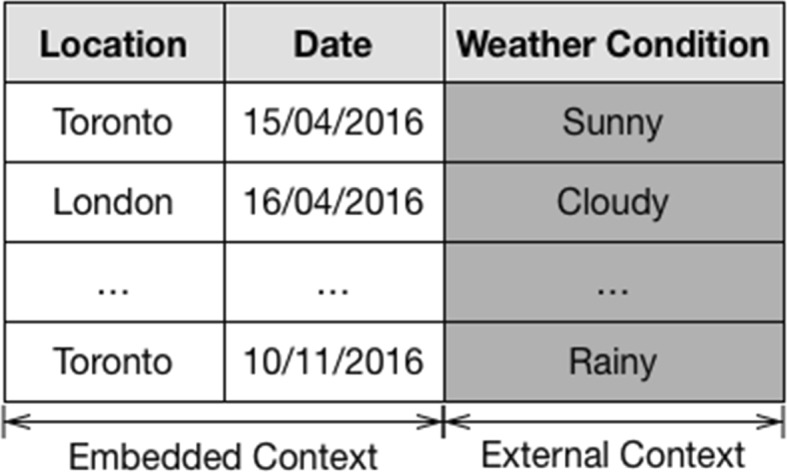
External service providing external contextual attributes

To exemplify such a data structure, Fig. 4 illustrates a scenario in which two contextual attributes embedded in the dataset (location and date) are used to match an external contextual value (weather condition).

The *contextual model storage* component functions as the interface between the *training phase* and the *production phase*, handling storage of and access to all the contextual collaborative filtering models trained during the *training phase*. These models will later be used by the *recommender engine* to provide customized *recommendations*.

### Training layer

The *training layer* contains the components required to extract contextual attributes from the rating data provided by the *rating storage* and to train *CF* models. These components are used to generate the contextual models that the *recommender engine* will use to provide personalized *recommendations* during the *production phase*.

This layer is separated into two components. The *contextual filtering* component is responsible for identifying the contextual attributes of past ratings and performing contextual inference when required. The *recommender trainer* component is responsible for carrying out training for each of the identified contextual attributes.

**Fig. 5:**
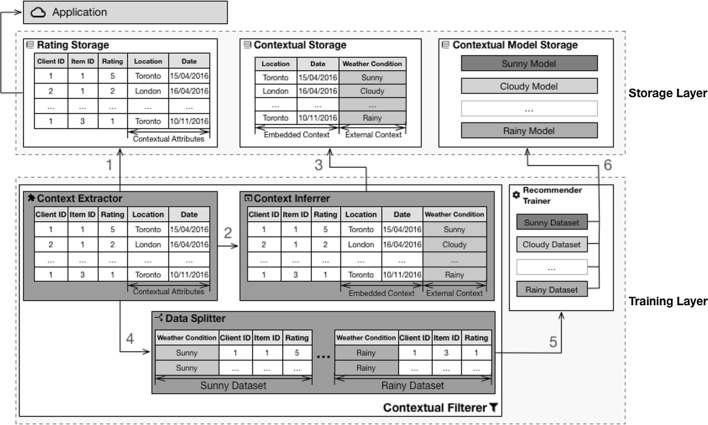
Training phase using the weather condition context

Figure 5 illustrates the steps followed by $({ CF})^2$ to train the models using weather condition as the contextual attribute. This illustration of the *training phase* is expanded as follows:

1. The starting point of the *training phase* occurs within the *context extractor* sub-component. This sub-component requests the *rating storage* component to provide a list of the ratings captured by the *application*. This list contains the *client identifier*, *item identifier*, *rating*, and contextual attributes *location* and *date*;
2. Because the weather condition cannot be retrieved directly from these attributes, the *contextual extractor* sub-component delegates the contextual inference task to the *context inferrer* sub-component;
3. To obtain the weather conditions at the time of a rating, the *context inferrer* sub-component must reach out to the *contextual storage* component. In this case, the *contextual storage* component provides a list containing the location and date followed by the weather condition;
4. With the list of contexts in place, the *context extractor* component invokes the *data splitter* sub-component to divide the rating dataset into smaller datasets, each of them representing a different context, in this case a weather condition;
5. For each contextual dataset, the *data splitter* sub-component invokes the *recommender trainer* component to train the local collaborative filtering models.
6. This results in the creation of a set of new models that will be stored in the *contextual model storage* component.

To accommodate new ratings captured by the *application* and to provide more up-to-date recommendations, this layer must be periodically invoked by an external system. The exact periodicity is domain-specific and must be addressed on a case-by-case basis.

### Production layer

The *production layer* is responsible for handling incoming requests made to the *recommender service* and returning the list of personalized *recommendations*.

This layer is divided into two components. The *request inspector* component is responsible for handling all incoming requests to identify their contextual attributes. The *recommender engine* component is responsible for choosing the proper model and for generating the *recommendations*.

**Fig. 6:**
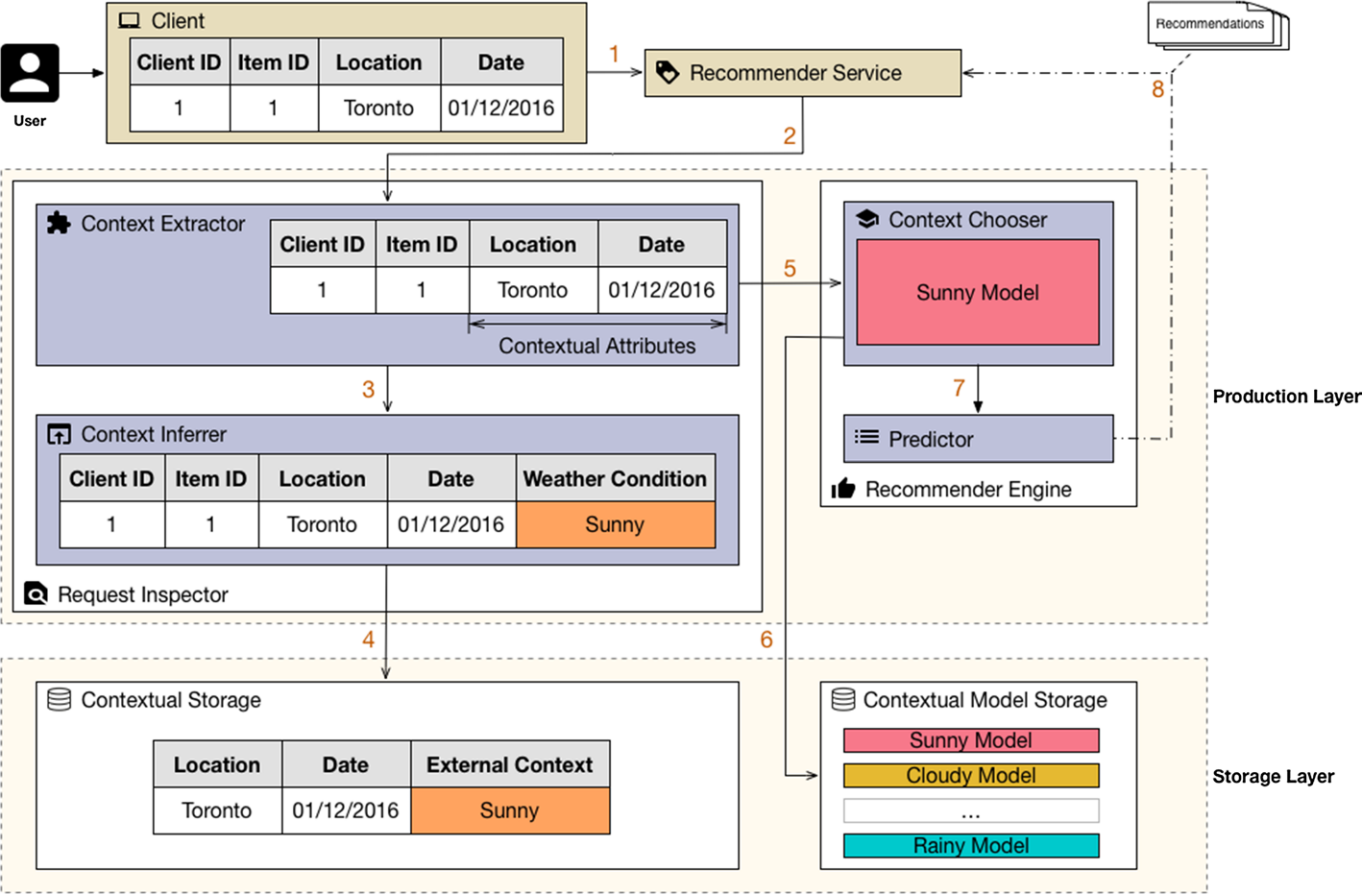
Production phase using the weather condition context

Figure 6 illustrates the steps followed by $({ CF})^2$ for an incoming request during the *production phase*. This illustration of the *production phase* is further described below.

1. The *production phase* starts every time a *client* issues a request to the *recommender service*. Using the example illustrated in Fig. 6, the request contains the *client identifier*, *item identifier*, and contextual attributes *location* and *date*;
2. The *recommender service* then delegates the request to the *context extractor* sub-component of the *request inspector* component;
3. The *context extractor* sub-component extracts the contextual attributes present in the request. In this example, the *contextual extractor* sub-component delegates the contextual inference task to the *context inferrer* sub-component;
4. Similarly to the training phase, the *context inferrer* sub-component reaches out to the *contextual storage* component to obtain the weather condition at the time of the request;
5. With the context properly identified, the *context extractor* sub-component invokes the *context chooser* sub-component of the *recommender engine* component. The purpose of this invocation is to obtain the corresponding contextual model;
6. The *context chooser* sub-component fetches the model from the *contextual model storage* component;
7. The retrieved model is then passed to the *predictor* sub-component, which queries the model for the list of recommendations;
8. This list of recommendations is then returned to the client.

## Evaluation

This section presents an evaluation of the $({ CF})^2$ architecture using two case studies. These case studies simulated the recommender system of a Web site. To train the collaborative filtering models, past interactions between clients and the Web site were used.

These past interactions were provided by a multi-media company specialized in weather- and traveller-related content and technology. Historical traffic was captured in clickstream form by two on-line marketing tools and Web analytics applications: *Google analytics*^4^ and *Adobe Omniture*.^5^ All data that contained pseudo-identifiers were collected in accordance with privacy policies. No personally identifiable information about users was used.

The task being evaluated is defined in the literature as *Find Good Items*. In this task, the recommender system is interested in suggesting items to a user, but displaying only those that are a “best bet”.

### Methodology

The evaluation scenario consists of a dataset *D* divided into two subsets, a training set *T* and a validation set *V*. The training set represents 80% of the dataset and is obtained by random selection from the original dataset without repetition. The remaining 20% represents the validation set.

Because an explicit rating is not provided by users, an implicit rating $r_{{ ui}}$ of 1 is used to indicate that a user *u* is interested in the requested page *i* when the user accesses the page. Moreover, because *CF* models need enough data to generate good recommendations, $r_{{ ui}}$ with contextual attributes representing less than 0.1% of the total dataset were removed.

**Fig. 7:**
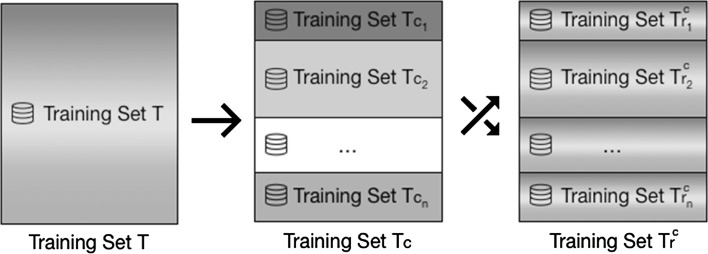
Training set *T* segmented by contextual attributes and random dataset reduction

Each contextual attribute *c* is represented by a training subset $T_c$ and a validation subset $V_c$ representing ratings of *T* and *V* containing the contextual attribute *c*. Moreover, because this research aims to prove that the use of contextual attributes as filtering criteria is better than random dataset reduction, the training subset $T_r^c$ and the validation subset $V_r^c$ represent randomly selected subsets of *T* and *V* with the same size as $T_c$ and $V_c$. This process is illustrated in Fig. 7. Each box expressing a Training Set $T_c$ is visualized by a unique color indicating that the set comprises of the entities with the same contextual attributes. On the other side, each box representing a Training set $T_r^c$ is depicted by mixed colors demonstrating the randomness of contextual attributes within the set.

Moreover, because the evaluation is performed by comparing the proposed architecture with the traditional approach, the subsets *T* and *V* represent the rating $r_{{ ui}}$ given by a user *u* to a page *i*, regardless of context. Both sets will be used to train and validate the traditional *CF* model *m*.

To assess the predictive quality of the models, this research used *predictive accuracy metrics*. The metrics used are the *mean squared error* (*MSE*) and *root mean squared error* (*RMSE*), which are given by the equation

1 $$\begin{aligned} \hbox {MSE(V)}= & {} \frac{1}{|V|} \cdot \displaystyle \sum _{(u,i) \in V}\left( r_{{ ui}}-\hat{r}_{{ ui}}\right) ^{2} \end{aligned}$$

2 $$\begin{aligned} \hbox {RMSE(V)}= & {} \sqrt{\frac{1}{|V|} \cdot \displaystyle \sum _{(u,i) \in V}\left( r_{{ ui}}-\hat{r}_{{ ui}}\right) ^{2}} \end{aligned}$$

where *V* is the validation set, |*V*| is the size of *V*, $r_{{ ui}}$ is the true user rating, and $\hat{r}_{{ ui}}$ is the predicted rating. These and other notations used throughout the section are presented in Table 1.

**Table 1:** Notations used throughout this paper

Symbol	Description
$r_{{ ui}}$	Rating given by a user *u* to page *i*
$\hat{r}_{{ ui}}$	Predicted rating given for a user *u* to page *i*
*D*	Dataset containing ratings given by a user *u* to page *i*
*T*	Subset of *D* containing random ratings and where $|T| = 0.8\cdot |D|$
$T_c$	Subset of *T* containing ratings given in a context *c*
$T_r^c$	Subset of *T* containing random ratings and where $|T^c_r| = |T_c|$
*V*	Set $D-T$
$V_c$	Subset of *V* containing ratings given in a context *c*
$V_r^c$	Subset of *V* containing random ratings and where $|V_r^c| = |V_c|$
*m*	*CF* model trained using set *T*
$m_c$	*CF* model trained using set $T_c$
$m_r^c$	*CF* model trained using set $T_r^c$

Training dataset *T* was then used to feed the training process by means of the *rating storage* component. This step ensures the creation of a different model $m_c$ for each contextual attribute *c* present in *T*, as illustrated in Fig. 3.

**Fig. 8:**
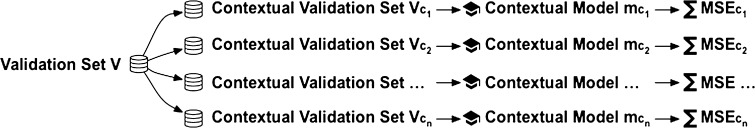
Validation set *V* segmented into subsets $V_c$ to predict ratings using model $m_c$

Similarly to the training dataset, validation is performed by splitting the validation set *V* into different contextual subsets $V_c$ and using these subsets to compare the predicted rating $\hat{r}_{{ ui}}$ with the rating $r_{{ ui}}$ available in the validation set $V_c$. This process is illustrated in Fig. 8.

To evaluate the models *m*, representing the traditional approach, and $m_r^c$ , representing random dataset reduction, the $({ CF})^2$ implementation was adapted to ignore context.

### Case studies

Because $({ CF})^2$ can use two types of contextual attributes, embedded and inferred, the evaluation process was divided into two case studies, each covering one of these types.

For the *CF* recommendation engine, both case studies used the *matrix factorization technique* with the *alternating least squares* (Koren et al. 2009) (*ALS*) algorithm. This has already been implemented by *Spark’s* *spark.mllib* machine learning library, providing an “out-of-the-box” solution that can process large volumes of data. Moreover, this implementation includes a training technique based on the work done by Hu et al. (2008), which specializes in training *CF* models using implicit ratings.

The model parameters were obtained after performing a cross-validation with partitions of the *T* dataset. Various configurations of the regularization parameter ($\lambda$), the number of hidden features, the number of iterations, and the confidence level ($\alpha$) were considered. The parameter values that resulted in a minimum stable *MSE* were chosen and are given in Table 2.

**Table 2:** Parameters used to train the *CF* models

Parameter	Value
$\lambda$ (regularization parameter)	100
Number of hidden features	20
Number of iterations	10
$\alpha$ (confidence level)	1500

Each case study was executed on a private server with a 24-core Intel Xeon E5-2630 2.3 GHz and 96 GB RAM DDR3 1600 MHz running Ubuntu 14.04.2 LTS.

#### Case study 1: embedded context

The first case study used clickstream data captured by the *Google Analytics* tool during the summer of 2016 (June 20 to September 22) to create recommender models based on the contextual attribute “operating system with platform”. To achieve this goal, the data were pre-processed to remove entries captured by the clickstream that did not represent a page view. These entries usually represent interactions with objects inside a Web page that do not trigger a page change, like interaction with map objects or social media snippets. After this step, the resulting dataset was filtered to contain only unique values with the following properties:

- Visitor identifier
- Uniform resource locator (URL)
- Operating System
- Platform is mobile (true or false)

This process resulted in a dataset containing 130,684,845 unique samples. The Number of unique users was 33,624,517, and the number of items (unique URLs) was 9,823,125. The final dataset was then split into a training set *T* and a validation set *V*.

The training set was then used to create the contextual models $m_c$, $m_r^c$, and *m*, and to calculate the time spent to train them. Moreover, the validation set was used to calculate the *MSE* of each model.

**Fig. 9:**
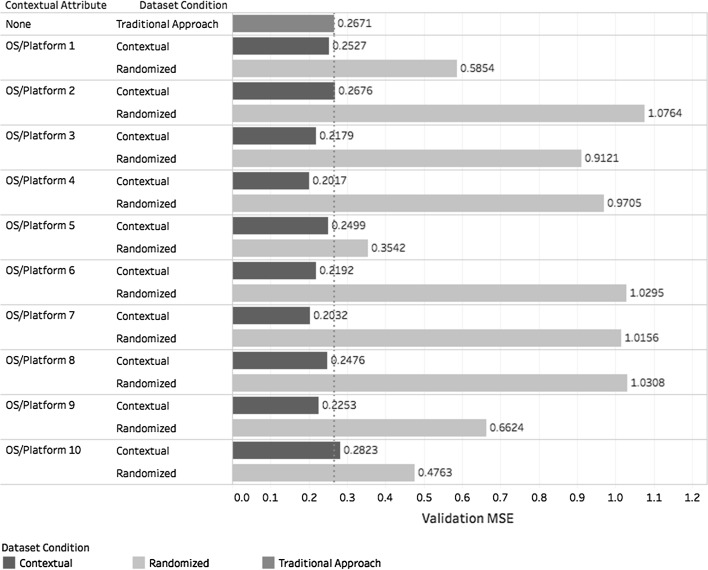
*MSE* of *m*, $m_c$, and $m_r^c$ segmented by dataset

The performance of the $({ CF})^2$ architecture is graphically represented in Fig. 9. For presentation purposes, all contextual names were converted to the format “OS/Platform”. The *MSE* obtained for the traditional approach, represented by model *m* was compared with each model $m_c$ and its equivalent model $m_r^c$ obtained by random reduction. To facilitate interpretation, the *MSE* for model *m* is displayed as a dotted reference line.

From Fig. 9, it is clear that using the proposed architecture generally provided better results than the traditional approach and significantly better results than random dataset reduction. This is also corroborated by the *MSE* and *RMSE* values obtained for each approach given in Table 3.

**Table 3:** *MSE* and *RMSE* obtained in case study 1

Approach	*MSE*	*RMSE*
Traditional	0.2671	0.5168
Contextual	0.2367	0.4865
Randomized	0.8113	0.9007

**Fig. 10:**
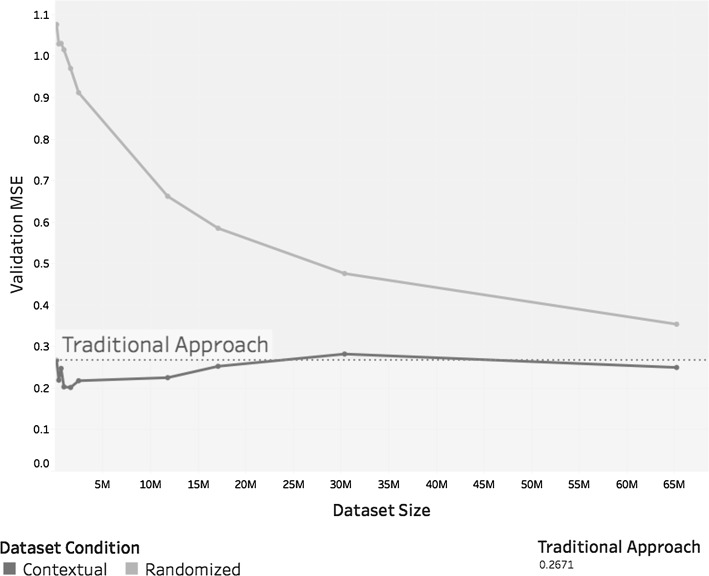
*MSE* value by dataset size for each dataset reduction technique

Figure 10 relates the *MSE* of each model with the dataset size. Analysis of Fig. 10 shows that when using contextual dataset reduction, the *MSE* values tend to be lower than the value obtained using the traditional approach, regardless of dataset size. The same is not true for random dataset reduction. When using this technique, the error increases as the dataset becomes smaller and converges to the accuracy of the traditional approach as the dataset increases in size. This is to be expected because there is almost no dataset reduction when the size approaches the original. The size of each dataset classified by contextual attribute is displayed in Table 4.

**Table 4:** Size of each dataset of case study 1 classified by contextual attribute

Contextual attribute	Dataset size		
	Full dataset	Training set	Validation set
OS/platform 1	17,099,871	13,681,058	3,418,813
OS/platform 2	151,710	120,902	30,808
OS/platform 3	2,464,423	1,971,104	493,319
OS/platform 4	1,623,950	1,300,121	323,829
OS/platform 5	65,221,859	52,179,154	13,042,705
OS/platform 6	385,096	307,823	77,273
OS/platform 7	923,827	738,438	185,389
OS/platform 8	626,817	501,195	125,622
OS/platform 9	11,812,359	9,452,167	2,360,192
OS/platform 10	30,374,933	24,300,878	6,074,055
Total	130,684,845	104,552,840	26,132,005

**Fig. 11:**
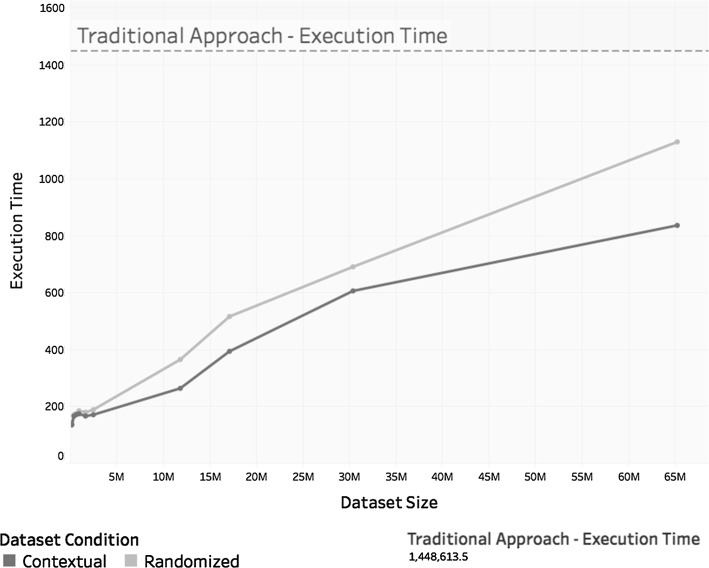
Training time in seconds taken to train each model

Furthermore, the times (in s) taken to train each model *m*, $m_c$, and $m_r^c$ were compared and are illustrated in Fig. 11.

These values indicate that the time to train each model increased almost linearly with dataset size. This was especially true in the case of *Spark’s* implementation of *ALS*, but other *matrix factorization* implementations should also experience a significant reduction in training time when dataset reduction is used.

#### Case study 2: contextual inference

The second case study used contextual inference to create recommender models based on the contextual attribute “weather condition”. This case study used click-stream data captured by the *Adobe Omniture* tool between April 1, 2015 and June 30, 2015 and included only visits generated by users in London, ON, Canada. The captured data contained unique entries with the following properties:

- Visitor identifier
- Uniform resource locator (URL)
- Time of access
- Location

After filtering out entries that did not represent a page view and removing duplicate entries, the resulting dataset contained 7,729,696 samples. Because the dataset still lacked the “weather condition” property, the *time of access* property along with the *location* property were used to obtain the weather condition for each visit. After removing duplicate entries, the dataset containing the tuples *visitor identifier*, *URL*, and *weather condition* was reduced to 3,181,808 entries. The Number of unique was users: 656,962 and the number of items ( unique URLs) was 3777. The final dataset was then split into a training set *T* and a validation set *V*.

**Fig. 12:**
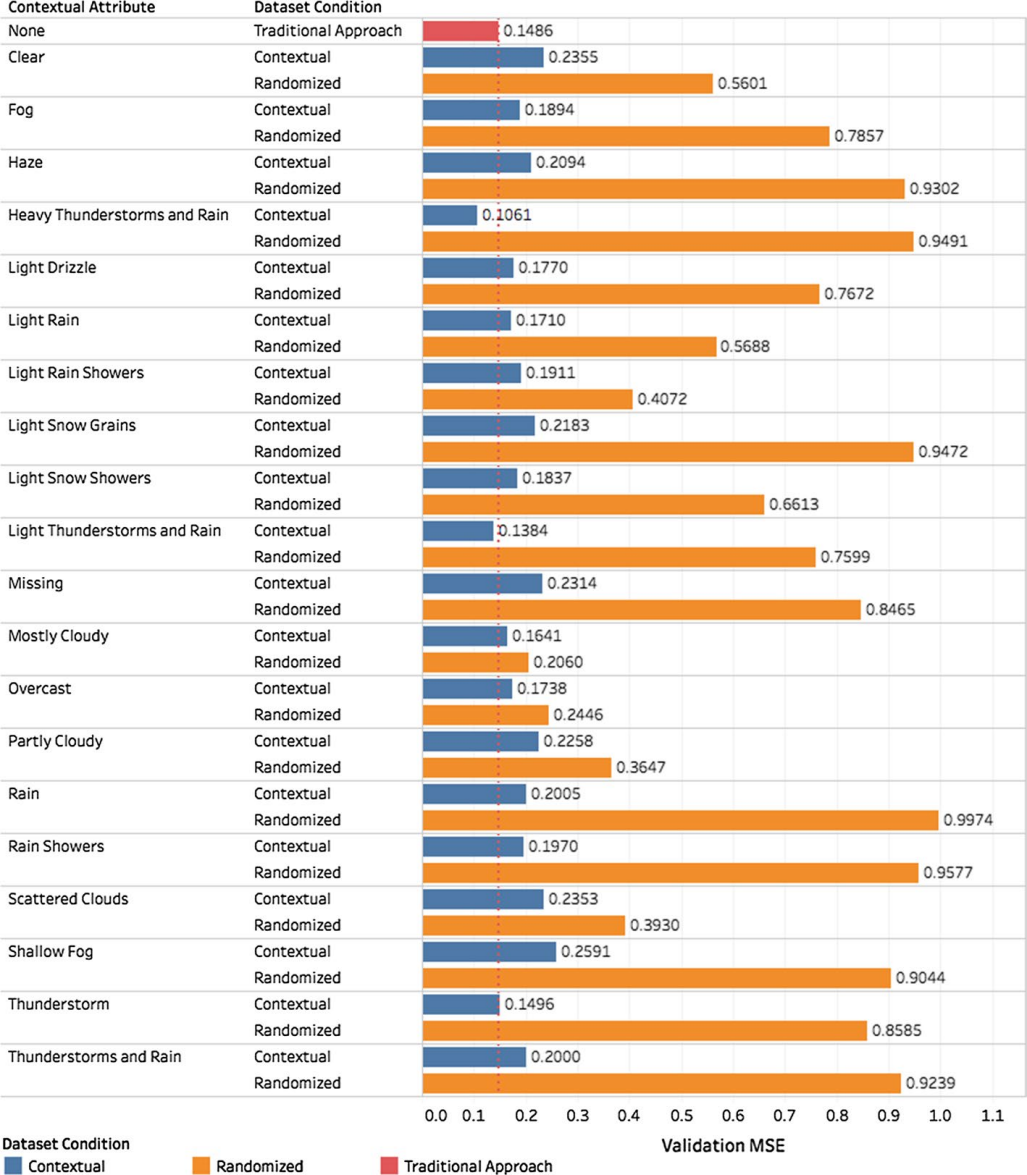
*MSE* of *m*, $m_c$, and $m_r^c$ segmented by dataset

The training set was then used to create the contextual models $m_c$, $m_r^c$, and *m* and to calculate the time spent to train them. Moreover, the validation set was used to calculate the *MSE* of each model, as shown in Fig. 12.

The *MSE* and *RMSE* values for each approach are given in Table 5.

**Table 5:** *MSE* and *RMSE* obtained in case study 2

Approach	*MSE*	*RMSE*
Traditional	0.1486	0.3855
Contextual	0.1977	0.4446
Randomized	0.7316	0.8553

Analysis conducted on these values indicates that $({ CF})^2$ yielded similar accuracy to the traditional approach while outperforming the accuracy obtained using random dataset reduction. Moreover, results indicated that accuracy improved as weather conditions deteriorated. The most compelling evidence was the *MSE* obtained for “heavy thunderstorms and rain” and “thunderstorm” weather conditions. This occurred because under these circumstances, rating patterns among users are shared. In other words, users have the tendency to check the same Web pages during bad weather.

Despite the fact that *MSE* improved in some contexts, the results obtained indicate that weather condition is a less suitable contextual attribute than “operating system with platform” for this application. Nevertheless, the use of a contextual attribute as a filtering criterion for local learning is more appropriate than random selection.

**Fig. 13:**
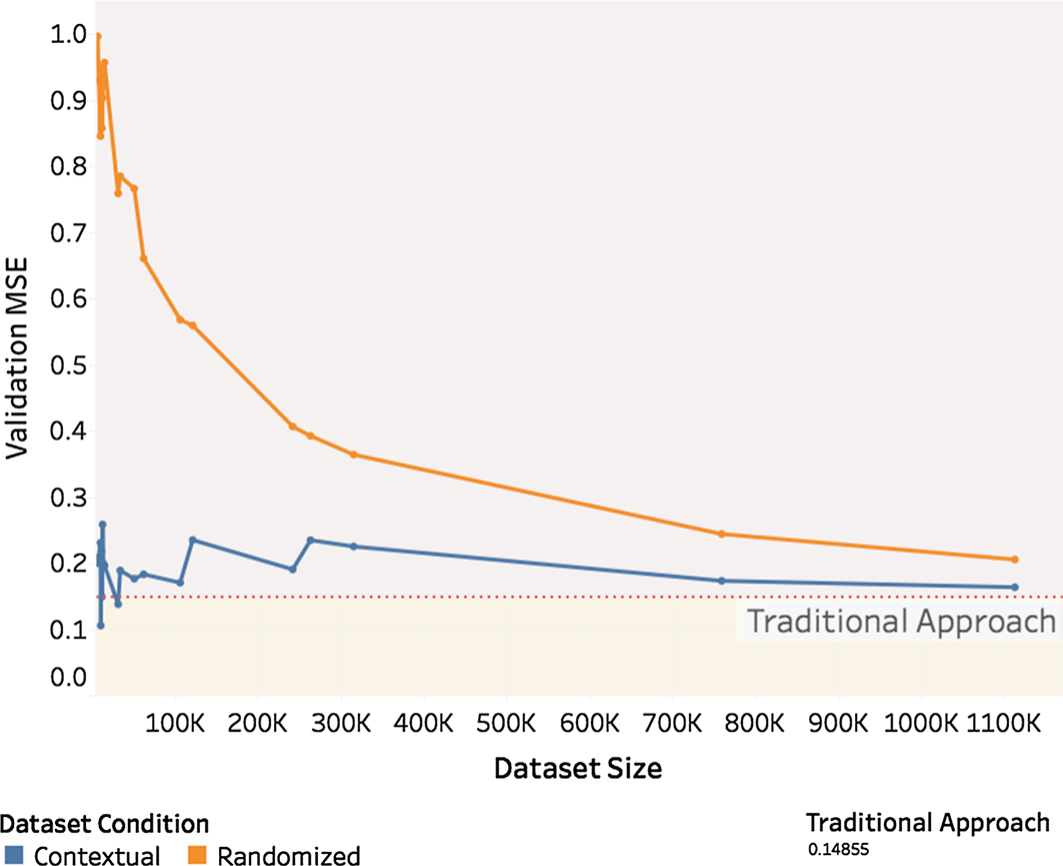
*MSE* value by dataset size for each dataset reduction technique

Taking dataset size into consideration, as shown in Table 6, this case study showed similar behaviour as the previous case study. Like the first case study, the second one showed that the *MSE* values tended to fluctuate around the value obtained for *m*, and both case studies showed that $m_c$ outperformed $m_r^c$. This situation is shown in Fig. 13, which also shows the *MSE* value for model *m* as a dotted reference line.

**Table 6:** Size of each dataset of case study 2 classified by contextual attribute

Contextual attribute	Dataset size		
	Full dataset	Training set	Validation set
Clear	120,430	96,511	23,919
Fog	32,945	26,437	6508
Haze	7241	5832	1409
Heavy thunderstorms and rain	9478	7564	1914
Light drizzle	49,873	40,000	9873
Light rain	105,339	84,186	21,153
Light rain showers	241,165	192,911	48,254
Light snowfall	10,159	8163	1996
Light snow showers	61,258	48,943	12,315
Light thunderstorms and rain	30,470	24,344	6126
Missing	9164	7315	1849
Mostly cloudy	1,112,850	890,189	222,661
Overcast	758,930	607,310	151,620
Partly cloudy	314,711	251,800	62,911
Rain	5719	4574	1145
Rain showers	14,002	11,216	2786
Scattered clouds	262,744	210,320	52,424
Shallow fog	11,711	9369	2342
Thunderstorm	10,979	8678	2301
Thunderstorms and rain	12,640	10,126	2514
Total	3,181,808	2,545,788	636,020

**Fig. 14:**
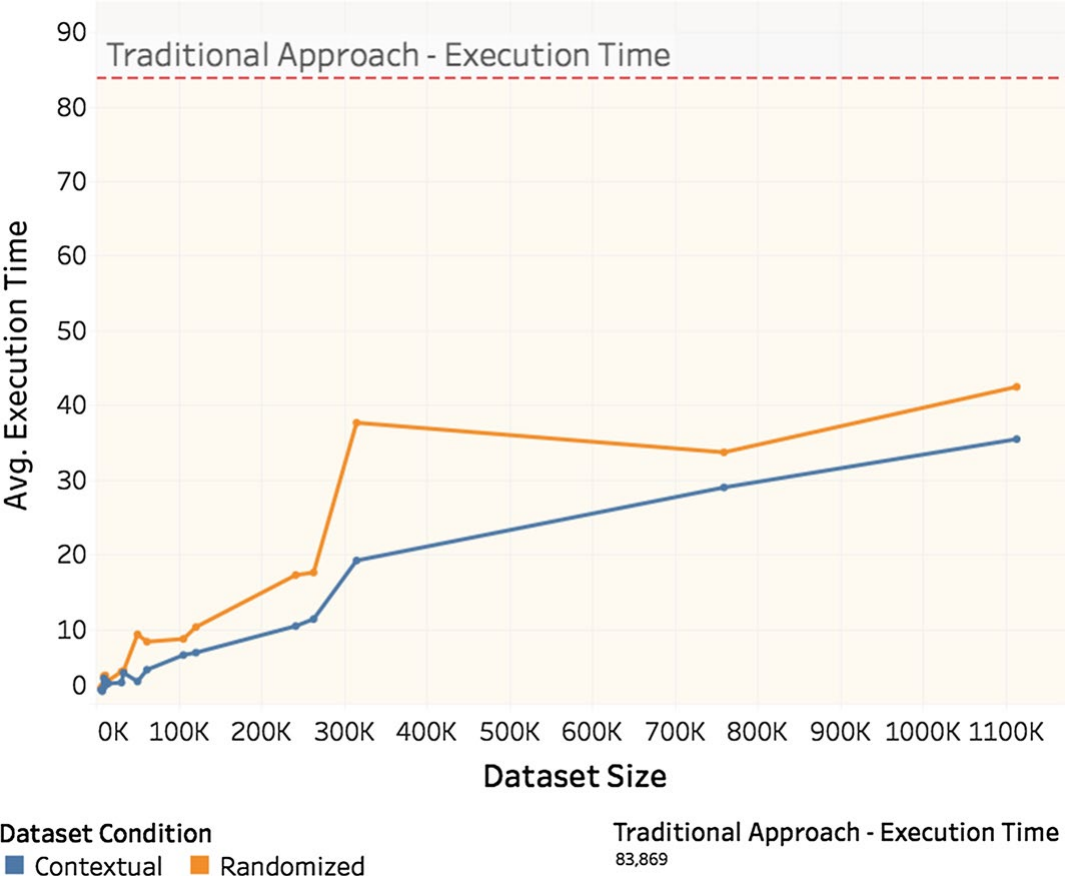
Time in seconds taken to train each model

Analysis of the time (in s) taken to train each model *m*, $m_c$, and $m_r^c$ were conducted, and the results are illustrated in Fig. 14. The results corroborate the findings of the previous case study and show that training time increases almost linearly with dataset size.

#### Discussion

The presented experiments show that the $({ CF})^2$ architecture achieves similar (or better) accuracy using a small fraction of the data to collaborative filtering models trained with the complete dataset. Whereas the second case study uses a single contextual attribute (weather), the first case study combines two attributes, operating system, and platform (mobile or not), into a single attribute OS/Platform?. When several attributes are involved, the amount of data may not be sufficient for training when data are split, and further investigation is needed to determine which attributes should be included. In the case study 1, combining attributes was possible as the dataset consisted of over 130 million records and after splitting, segments still remained sufficiently large.

Both studies used the categorical attributes; nevertheless, handling continuous attributes can be done by converting them into categorical ones according to ranges. Depending on the attributes, selecting different ranges may result in different recommendation accuracies.

In the first case study, the proposed approach provided better recommendations then the traditional one (Table 3) whereas, in the second one, the traditional approach was slightly better (Table 5). Observing Fig. 12, it can be noticed that the $({ CF})^2$ accuracy improved as condition deteriorated: accuracy was much better with $({ CF})^2$ than with the traditional approach for ”heavy thunderstorms and rain”. The results indicate that the choice of the contextual attribute as well as the actual values of the attributes impact the quality of recommendations. Therefore, the important direction of future research in context-aware recommendation systems is a selection of context attributes to include in the recommendation system.

## Conclusions

The work described in this paper has developed the $({ CF})^2$ architecture, which uses local learning techniques to embed contextual awareness into *CF* models. By incorporating context into standard *CF* models, recommender applications can provide recommendations with a better chance of being relevant to users. Moreover, local learning using context as a filtering criterion enables the use of large datasets in recommender systems. An overview of the architecture has been presented, including its components, their roles, and their relationships.

Because of the generic approach of $({ CF})^2$, its implementations can incorporate context-aware recommender systems using one of the several widely available collaborative filtering libraries.

The proposed architecture has been demonstrated on the two case studies involving Find Good Items task. The first case study used embedded context, and the second used external knowledge by means of contextual inference. Both case studies involved large real-world datasets, were evaluated using the same methodology, and their accuracy was compared against the traditional method. The results indicate that contextual models trained with a small fraction of the data gave similar accuracy to models trained with the full dataset. To explore impact of context on large dataset models, local models created using random sampling were compared to contextual dataset reduction, and the results demonstrated that using contextual information outperforms random selection in accuracy.

Extensions of this research will consider using instance selection techniques to reduce dataset sizes even further. Moreover, privacy and security issues associated with recommender systems can be incorporated into the $({ CF})^2$ architecture.

## Footnotes

1 http://www.amazon.com.

2 http://www.tivo.com.

3 http://www.netflix.com.

4 http://google.com/analytics.

5 http://www.omniture.com.
